# Xeroform gauze versus silver sulfadiazine for mixed-depth pediatric scald injuries: A retrospective study

**DOI:** 10.1016/j.jpra.2024.11.006

**Published:** 2024-11-16

**Authors:** Dominic Alessio-Bilowus, Justin D Klein, Elika Ridelman, Christina M Shanti

**Affiliations:** Department of Surgery, Division of Pediatric Surgery, Children's Hospital of Michigan/Wayne State University School of Medicine, Detroit, MI, USA

**Keywords:** Scald burn, Pediatric, Xeroform, Silver sulfadiazine, Skin graft

## Abstract

**Introduction:**

Silver sulfadiazine 1 % cream had historically been the mainstay initial treatment for scald wounds at our institution. However, we transitioned to using closed dressings of only petrolatum-impregnated 3 % bismuth tribromophenate gauze (Xeroform) for all partial-thickness burns. Xeroform adheres to the wound while allowing the exudates to drain and acts as a scaffold for re-epithelialization, after which it falls off without traumatizing the wound bed, theoretically allowing viable tissue to declare itself while requiring less frequent dressing changes.

**Materials and Methods:**

A retrospective chart review was conducted of patients aged ≤5 years with mixed-depth scald injuries between the years 1) 2004 and 2008, during which silver sulfadiazine was the standard initial choice and 2) 2015 and 2018, when only Xeroform was used as the standard.

**Results:**

The study included 347 patients, among whom 200 were treated with silver sulfadiazine and 147 were treated with Xeroform alone. The 2 groups had similar burn sizes and rates of skin grafting (silver group 30/200 [15.0 %] and Xeroform group 20/147 [17.7 %]) However, the Xeroform group showed longer time from injury to grafting (24 vs. 9.9 days, *p* = 0.002) but showed a significantly smaller mean graft size than the silver group (147 vs. 336 cm^2^, *p* = 0.027).

**Conclusions:**

These findings suggest that using Xeroform may promote better wound healing than using silver sulfadiazine. In addition, patients with Xeroform can be discharged with their dressings in place for grafting in the outpatient setting, during which time they are in closed dressings without frequent changes and associated discomfort.

## Introduction

Burn injuries are ubiquitous, affecting millions of people globally and leading to significant morbidity and even fatality. Although the rates of injury and severity are comparatively lower in high-resource countries owing to safety regulations and cultural differences, burns continue to represent a major source of trauma in the United States. A significant proportion of these cases involve children, with patients aged <16 years accounting for 23 % of acute burn admissions in the US and 58 % of this group falling below the age of 5 years.^1^^,^^2^ Fortunately, advances in care strategies and technology have resulted in improved outcomes in the 21st century.^3^

One of the central components of any burn care plan has always been the method of selecting and applying a wound dressing, for which a host of options are available. Historically, natural substances such as honey, tilapia skin, or banana leaves have been used, whereas more contemporary products include biological dressings such as porcine xenografts, amniotic membranes, and biosynthetic membranes.^3^, ^4^, ^5^, ^6^ However, one of the most widely used methods since the second half of the 20th century involved the use of silver-containing ointments such as silver sulfadiazine 1 % cream, commonly sold under the trade name Silvadene® (Pfizer, New York, NY).^3^^,^^7^ Aside from its moisturizing and protective qualities, silver sulfadiazine cream gained support owing to the antimicrobial properties of silver as a heavy metal, in particular against *Pseudomonas* species, although its effectiveness has been debated alongside concerns regarding silver absorption or other side effects.^8^, ^9^, ^10^, ^11^, ^12^, ^13^

Accordingly, silver sulfadiazine 1 % cream was considered the mainstay initial treatment for burn wounds at our Level 1 pediatric burn unit for decades. However, over the last several years, we gradually discontinued its use and transitioned to closed dressings of Xeroform gauze (Curad®, Medline Industries, Northfield, IL) in the initial care of partial-thickness burns, including those with heterogeneous depth such as scald injuries, with the goal of reducing patient discomfort and improving healing. Xeroform, which is a petrolatum-soaked gauze dressing containing 3 % bismuth tribromophenate as a heavy metal antimicrobial agent, can be placed directly on the tissue so that the base layer remains on the wound for up to 3 weeks. Silver-based creams typically require daily dressing changes, which are associated with significant patient discomfort. More importantly, we hypothesized that the mechanical forces of constant wound cleaning could harm the healing tissue resulting in poorer outcomes, especially in the zone of stasis in mixed-depth burns.^14^ Over time, the zone of stasis demarcates into more clearly viable or non-viable tissue, guiding decisions on excision and grafting.^15^^,^^16^ However, this zone is especially at risk of mechanical damage, potentially resulting in disruption to the healing process.

The purpose of this study was to compare the outcomes of our patients who were treated with the initial use of Xeroform gauze to the patients who were treated with silver sulfadiazine cream, with respect to skin grafting.

## Methods

After being granted appropriate institutional review board approval, a retrospective chart review was conducted of all patients aged ≤5 years who presented to our urban, academic children's hospital with mixed-depth scald injuries within 2 distinct timeframes: 1) between the years 2004 and 2008, during which silver sulfadiazine 1 % cream was the standard initial choice for wound care and 2) between the years 2015 and 2018, when Xeroform became the standard initial dressing. To reduce bias, the period between 2009 and 2014 was excluded, since selection of the agent during this time was practitioner dependent. Electrical, chemical, grease, direct flame, and contact burns were excluded, as well as any burn covering <5 % of the total body surface area (TBSA). Data collected included patient demographics, percent TBSA burned, site of the burn, type of wound dressing used, length of hospital stay, any excision and grafting, time to grafting, graft size, and any postoperative complication including wound infection, which was defined by the documented clinical examination. All data were collected and entered manually into Microsoft Excel software. Analysis was conducted using IBM SPSS Statistics V22.0. The student's *t*-test was performed to evaluate the differences between groups, and significance was set at a standard p-value of 0.05 for all factors.

## Results

**Figure 1:**
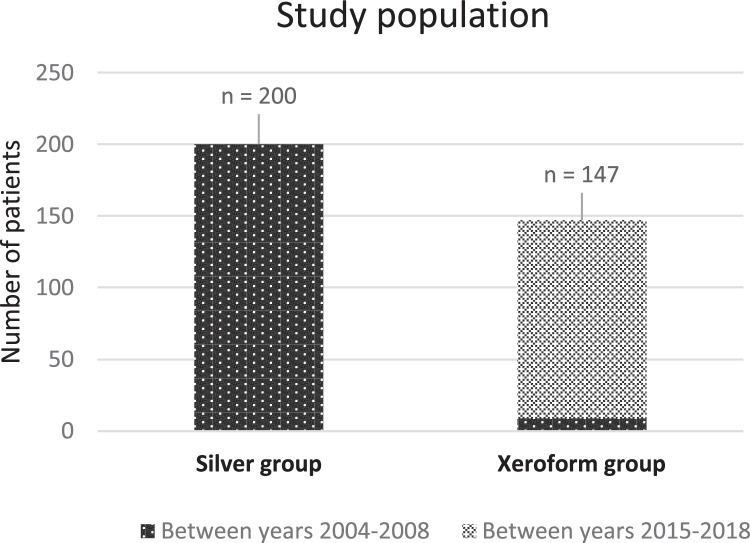
Study population. All 200 patients who received silver sulfadiazine cream were treated between the years 2004 and 2008. Among the 147 patients who received only Xeroform gauze, 9 were treated during the older time period and 138 during the more recent time period of 2015–2018.

The study included 347 patients in total, 200 of whom were treated with silver sulfadiazine 1 % cream (referred to as the “silver group”) and 147 were treated with Xeroform gauze (referred to as the “Xeroform group”). Demographics and burn characteristics of the study cohort are presented in (Table 1). In the silver group, all 200 patients were treated within the early time period between the years 2004 and 2008. A minority (29 %) of the patients within the silver group, in particular those with more severe burns that required grafts, also received some form of gauze wrapping between the silver sulfadiazine cream and the outer bandages during their hospital stay. However, the method followed for dressing changes when using silver sulfadiazine cream as the initial base treatment was consistent regardless of whether any additional overlying gauze was involved, leading us to consider all patients who were treated with silver cream in aggregate as the most clinically relevant grouping. Among the 147 patients within the Xeroform group, 138 were treated within the more recent time period of 2015–2018 whereas 9 patients were included from the older time period. All 147 patients within this group received only Xeroform gauze with no silver sulfadiazine cream (Figure 1).

**Table 1:** Study cohort demographics and burn characteristics. Descriptive statistics of the study cohort.

	Silver group (%) *n* = 200	Xeroform group (%) *n* = 147	Overall (%) *n* = 347
Mean age (years)	1.9	1.5	1.7
Sex			
Male	117 (58.5 %)	74 (50.3 %)	191 (55.0 %)
Female	83 (41.5 %)	73 (49.7 %)	156 (45.0 %)
Race			
White	34 (17 %)	41 (27.9 %)	75 (21.6 %)
Black	125 (62.5 %)	93 (63.3 %)	218 (62.8 %)
Hispanic	6 (3.0 %)	5 (3.4 %)	11 (3.2 %)
Other/unknown	35 (17.5 %)	8 (5.4 %)	43 (12.4 %)
Site of burn^a^			
Head and neck	84 (42.0 %)	60 (40.8 %)	144 (41.5 %)
Trunk	134 (67 %)	107 (72.8 %)	241 (69.5 %)
Pelvic region	32 (16.0 %)	31 (21.1 %)	63 (18.2 %)
Upper extremity	95 (47.5 %)	83 (56.5 %)	178 (51.3 %)
Lower extremity	82 (41.0 %)	61 (41.5 %)	143 (41.2 %)
Mean TBSA (%)	10.3 %	9.9 %	10.1 %
Skin graft used	30 (15.0 %)	26 (17.7 %)	56 (16.1 %)

a Site of burn includes patients with multiple sites of injury; thus, the percentages do not add up to 100 %. TBSA, total body surface area.

**Figure 2:**
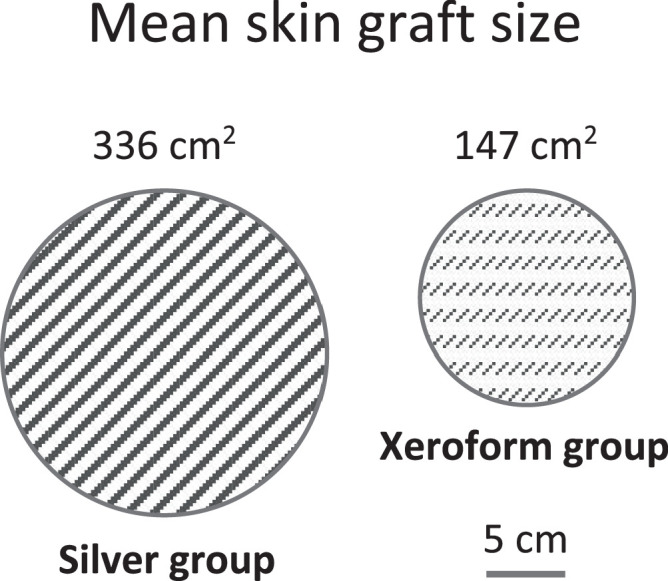
Mean skin graft size. The mean area of the skin grafts in each group is visually represented here for comparison.

The size of burns between the 2 groups was similar, with a mean burn TBSA of 10.3 % and 9.9 % in the silver and Xeroform groups, respectively. Rates of skin grafting were also similar, with 30 of 200 patients (15.0 %) receiving skin grafting in the silver group and 26 of 147 patients (17.7 %) in the Xeroform group receiving a graft. However, the mean area of the skin graft was significantly smaller among patients in the Xeroform group (147 cm^2^) than those in the silver group (336 cm^2^), representing a 56 % smaller graft size in the Xeroform group (*p* = 0.027) (Figure 2).

The length of time from injury to skin grafting was significantly longer in the Xeroform group at a mean of 24.0 days than 9.9 days in the silver group (*p* = 0.002). This finding was not affected by the incorporation of any time gap between the initial injury and hospital admission, with the number of days from admission to grafting in the Xeroform group at a mean of 23.8 days compared with 9.6 days in the silver group (*p* < 0.001). Further investigation revealed that a larger proportion of patients in the Xeroform group (18.4 %) returned for skin grafting in the outpatient setting, with this rate being only 10.0 % for the silver group (*p* = 0.024). Lastly, 10.2 % of the Xeroform group returned more than once, with only 2.5 % returning in the silver group (*p* = 0.002). There was no statistically significant difference in the mean length of hospital stay between the 2 groups (overall 7.2 days, Xeroform group 8.2 days, and silver group 6.5 days; *p* = 0.140) Furthermore, there was no statistical significance between the groups in the anatomic site of the burn or rate of complications, including wound infection.

## Discussion

Despite the 10-year time span between the patient groups, the fact that the burn TBSA remained approximately the same suggests no major changes in the mechanism and severity of scald burns in our local region in recent decades. Likewise, the similarity in the percentage of patients who received skin grafting lends support to the validity of the results and makes it less likely that the analysis was skewed by variations in the severity of the wound or injury pattern between the 2 groups.

The notable difference in the mean size of the skin graft between the 2 groups is consistent with our initial hypothesis that using only Xeroform gauze as the initial dressing for these mixed-depth scald burns may reduce damage to the at-risk zone of stasis compared with using silver sulfadiazine cream. The suggested mechanism for this benefit lies in the fact that Xeroform adheres to the wound while allowing the exudates to drain through and acts as a scaffold for re-epithelialization, after which it falls off without traumatizing the healed burn. Unlike ointments, the base layer of a Xeroform gauze dressing can remain on the wound for up to 3 weeks. The patient may bathe without removing the gauze, and even if the dressing is soiled, it can be washed gently with soap and water.

This characteristic protects the healing tissue within the zone of stasis from being stripped away during dressing changes, allowing the viable tissue to declare itself without disruption. By the time the Xeroform falls away on its own days to weeks later, or is removed in the operating room, the viable portions of the zone of stasis become clearly demarcated, allowing the surgeon to plan their excision more accurately and conservatively. This could result in smaller areas requiring grafting and smaller donor sites, which allows for more rapid healing, less chance of graft failure, and lower patient morbidity. This is in addition to the decrease in patient discomfort associated with the regular or daily dressing changes that are required when using silver sulfadiazine cream, even if it is used in combination with other gauze dressings. Other aspects of silver sulfadiazine cream that could potentially affect the graft size are the documented cytotoxic qualities of silver and risk of moisture-related tissue maceration, which could contribute to tissue damage.^10^

The increase in the time elapsed between injury and grafting in the Xeroform group, accompanied by a higher rate of return visits, is at least partially explained by the larger number patients returning for grafting in the outpatient setting rather than undergoing excision and grafting during their initial hospital stay. This delayed grafting, facilitated by the longevity of Xeroform gauze dressing, allows the viable tissue within the zone of stasis to declare itself more clearly as described above, which could result in smaller graft requirements. This also corresponds to the Xeroform's approximate lifespan of 3 weeks, which could be related to the 24-day timeline observed in the Xeroform group in our study. Importantly, the longer time prior to grafting does not place the patients in undue discomfort because they can return home with their Xeroform dressings in place and are only required to change the outer dressings. We recognize that the significant decrease in the size of skin grafts must also be interpreted in the context of the increased time to grafting and number of graft operations, and may, at least in part, represent the changes in surgeon practice or even national standards of care considering the time gap between the patient groups being compared.

Finally, the fact that our study did not show any statistically significant findings related to wound infection between the 2 groups is unsurprising considering that the previously published data have been inconclusive on the true clinical effect of the antiseptic components within silver sulfadiazine cream and Xeroform gauze.^12^^,^^13^^,^^17^ Thus, this study focused on the mechanical considerations of changing wound dressings as opposed to their antimicrobial properties or infection rates.

This study does have some inevitable limitations. First, our findings do not control for the differences in burn depth since all burns in this study were of mixed depth, as is expected of scald burn injuries which are heterogeneous in nature. Second, we recognize that the long-term outcomes such as scarring or time to healing merit discussion, but we could not explore these variables due to the lack of sufficient digitized clinical documentation from our older cohort (years 2004–2008). However, we are confident that our findings are clinically relevant and we hope to stimulate discussion among burn researchers and clinicians about wound dressing practice toward the advancement of pediatric burn care.

In conclusion, when comparing patients with mixed-depth scald burn treated with only Xeroform to those treated with silver sulfadiazine cream, the Xeroform group showed a longer time from injury to skin grafting but a mean graft size less than half of that in the silver group. Xeroform remains on a wound bed for days to weeks, whereas silver sulfadiazine cream must be changed regularly, which results in constant disruption of the healing tissue, in particular the at-risk zone of stasis. Fewer dressing changes combined with later skin grafting could reduce the mechanical forces on the healing tissue, which allows burn wounds to demarcate and heal more effectively, decreasing the size of the required skin grafts and donor sites. Although time to grafting was longer when using the gauze, these patients were in closed dressings with fewer changes and lesser discomfort.

## References

[1] Jeschke M.G., van Baar M.E., Choudhry M.A. (2020). Burn injury. Nat Rev Dis Primers.

[2] American Burn Association. National burn repository: report of data from 2009 to 2018. Retrieved from: https://sk75w2kudjd3fv2xs2cvymrg-wpengine.netdna-ssl.com/wp-content/uploads/2020/05/2019-ABA-Annual-Report_FINAL.pdf

[3] Jeschke M.G., Herndon D.N (2014). Burns in children: standard and new treatments. Lancet.

[4] Aziz Z., Abdul Rasool Hassan B (2017). The effects of honey compared to silver sulfadiazine for the treatment of burns: a systematic review of randomized controlled trials. Burns.

[5] Lima E.M., Moraes Filho M.O., Forte A.J. (2020). Pediatric burn treatment using tilapia skin as a xenograft for superficial partial-thickness wounds: a pilot study. J Burn Care Res.

[6] Gore M.A., Akolekar D (2003). Evaluation of banana leaf dressing for partial thickness burn wounds. Burns.

[7] Vloemans A.F., Hermans M.H., van der Wal M.B. (2014). Optimal treatment of partial thickness burns in children: a systematic review. Burns.

[8] Fox C.L (1968). Silver sulfadiazine-a new topical therapy for Pseudomonas in burns. Arch Surg.

[9] Fuller F.W (2009). The side effects of silver sulfadiazine. J Burn Care Res.

[10] Wasiak J., Cleland H (2015). Burns: dressings. BMJ Clin Evid.

[11] Rashaan Z.M., Krijnen P., Klamer R.R. (2014). Nonsilver treatment vs. silver sulfadiazine in treatment of partial-thickness burn wounds in children: a systematic review and meta-analysis. Wound Repair Regen.

[12] Vermeulen H., van Hattem J.M., Storm-Versloot M.N. (2007). Topical silver for treating infected wounds. Cochrane Database Syst Rev.

[13] Storm-Versloot M.N., Vos C.G., Ubbink D.T. (2010). Topical silver for preventing wound infection. Cochrane Database Syst Rev.

[14] Jackson D.M (1953). The diagnosis of the depth of burning. Br J Surg.

[15] Jackson D.M (1969). Second thoughts on the burn wound. J Trauma.

[16] Shupp J.W., Nasabzadeh T.J., Rosenthal D.S. (2010). A review of the local pathophysiologic bases of burn wound progression. J Burn Care Res.

[17] Barillo D.J., Barillo A.R., Korn S. (2017). The antimicrobial spectrum of Xeroform®. Burns.

